# Remote Safety Monitoring for Elderly Persons Based on Omni-Vision Analysis

**DOI:** 10.1371/journal.pone.0124068

**Published:** 2015-05-15

**Authors:** Yun Xiang, Yi-ping Tang, Bao-qing Ma, Hang-chen Yan, Jun Jiang, Xu-yuan Tian

**Affiliations:** 1 College of Information Engineering, Zhejiang University of Technology, Hangzhou, China; 2 College of Computer Science, Zhejiang University of Technology, Hangzhou, China; Xiamen University, CHINA

## Abstract

Remote monitoring service for elderly persons is important as the aged populations in most developed countries continue growing. To monitor the safety and health of the elderly population, we propose a novel omni-directional vision sensor based system, which can detect and track object motion, recognize human posture, and analyze human behavior automatically. In this work, we have made the following contributions: (1) we develop a remote safety monitoring system which can provide real-time and automatic health care for the elderly persons and (2) we design a novel motion history or energy images based algorithm for motion object tracking. Our system can accurately and efficiently collect, analyze, and transfer elderly activity information and provide health care in real-time. Experimental results show that our technique can improve the data analysis efficiency by 58.5% for object tracking. Moreover, for the human posture recognition application, the success rate can reach 98.6% on average.

## Introduction

As the life expectation around the world increases, the aging population problem is becoming more and more urgent and significant. According to the prediction of the United Nations, 65 years and older will occupy 15.7 percent of the total population in 2030 [1]. Huge amounts of resources are required for the government and society to take care of the aged population. On the other hand, it is unrealistic for the family members to provide daily and constant care, which is essential for the safety and health of the elders. Thus, remote monitoring systems which can provide health care services for the elderly persons living alone without the presence of family members are becoming popular and mainstream.

Recently, remote health monitoring research has drawn significant attention and resources from university researchers, corporations, and governments in many developed countries [2, 3]. Various cameras or sensors based monitoring systems [4, 5, 17] are developed to provide real-time safety and health monitoring and automatic abnormal behavior analysis for the elderly population. However, the conventional methods, which utilize single or multiple vision cameras or sensors, suffer from the problems of blind spot and system complexity.

Unlike other conventional surveillance systems, in elderly monitoring application, blind spot can be dangerous and life-threatening. Thus, it is imperative that we obtain omni-directional views of the entire house. Single sensor or camera is typically insufficient. However, deploying multiple sensors can make the system overly complex and unreliable. Therefore, we propose to use omni-directional vision sensor (ODVS) based monitoring system, which is omni-directional, simple, and inexpensive [18].

In our system, an ODVS device is installed in the room to provide a 360° panoramic image. To reduce the cost, provide daily care and surveillance, and raise alarms when abnormalities occur, it is important to process the derived images automatically and real-timely. However, although omni-directional images can provide an overview of the entire room, unlike conventional images, they can not be processed directly. Thus, we have developed a calibration technique to map the coordinates of the ODVS image to the actual locations inside the house. Based on the calibration technique, we propose novel motion tracking and posture recognition techniques to identify the status of the elderly persons. The life patterns and abnormalities of the elderly persons can be generalized and detected by analyzing the postures and behaviors. Once abnormalities are detected, the system would notify the persons immediately.

In general, we have made the following contributions:

1. we design and implement an accurate and efficient remote safety monitoring system based on the ODVS images;
2. we develop a novel motion history or energy image (MHoEI) based motion tracking algorithm, which can improve the motion tracking efficiency significantly.

Experimental results show that, based on our ODVS image calibration technique, our motion tracking technique can improve the tracking efficiency by 58.5%, and our posture recognition technique has a success rate of 98.6%.

## Related Work

Based on the signal acquisition methods, elderly health monitoring techniques can be generalized into three categories: physiological sensors based, environment monitoring sensors based, and image sensors based.

1. Physiological sensor based techniques. Elderly persons’ physiological conditions, such as ECG, blood pressure, respiration, blood glucose, and body temperature etc., are acquired using physiological sensors [19–22]. This data can be used to monitor the chronic diseases and diagnose the early symptoms. However, it is inconvenient and sometimes impossible for the elderly persons to wear those sensors all the time, which can greatly limit the effectiveness of this approach.
2. Environment monitoring sensors based techniques. The water, gas, and electricity usage activities are monitored using human activity sensors, switch sensors and flow sensors installed in the house [23]. By analyzing the collected data, the behavior patterns of the elderly persons are generalized and used to identify abnormal activities. The problem for this approach is that it is not guaranteed to report abnormalities in real-time. This can be dangerous because emergency occurs.
3. Image sensors based techniques. The images, captured by cameras and sensors, are transmitted back and analyzed accordingly using various image processing and data mining techniques. Researchers have proposed to use video streams [24, 25] to record the daily activities of the patients. However, because of the blind spots of the video cameras, the tracking of human motions can be easily lost. To address this problem, Lin et al. [26] have developed an intelligent surveillance system using an Omni-directional CCD Camera. However, because of the properties of the omni-directional images, the motion tracking algorithm becomes too complicated to be implemented in many real-world applications. In this work, we address those problems by proposing novel calibration, motion tracking, and posture recognition techniques.

Video or image based objective tracking and pattern recognition techniques are widely used in many applications [11–16]. Thus, the ODVS based technique has great potential to be implemented in other area, e.g., mobile visual search and augmented reality etc. In general, the objective tracking and pattern recognition techniques in the existing literature can be summarized into three categories: (1), point based [6]; (2), contour based [7, 8, 10]; and (3), kernel based [9]. The performance of the techniques vary according to the environment and applications. However, in our elderly monitoring application, it is more desirable and realistic for the system to run on an embedded platform. Thus, while satisfying the performance requirement, reducing the computational cost of the tracking and recognition algorithms should be priority. Thus, the contour based approaches is best suited to our system because of its low complexity and hence computational requirement. The most commonly used algorithm for the contour based solutions is the Camshift algorithm [7]. However, this algorithm suffers from the problem that it can not track and detect static objects. In this work, we have proposed a MHoEI based algorithm, which can detect static objects and are more efficient since we have used a smaller sampling window size.

## Methods

### System Design

Our goal is to design an affordable elderly safety monitoring system which can detect abnormal behaviors and protect patient privacy simultaneously. The system should also be able to alarm the corresponding agencies or family members when emergency occurs. Moreover, authorized persons should be able to monitor the daily activities of the elderly persons remotely under their consent. The status of the elderly person is described using time, environment, posture, and action.

**Fig 1:**
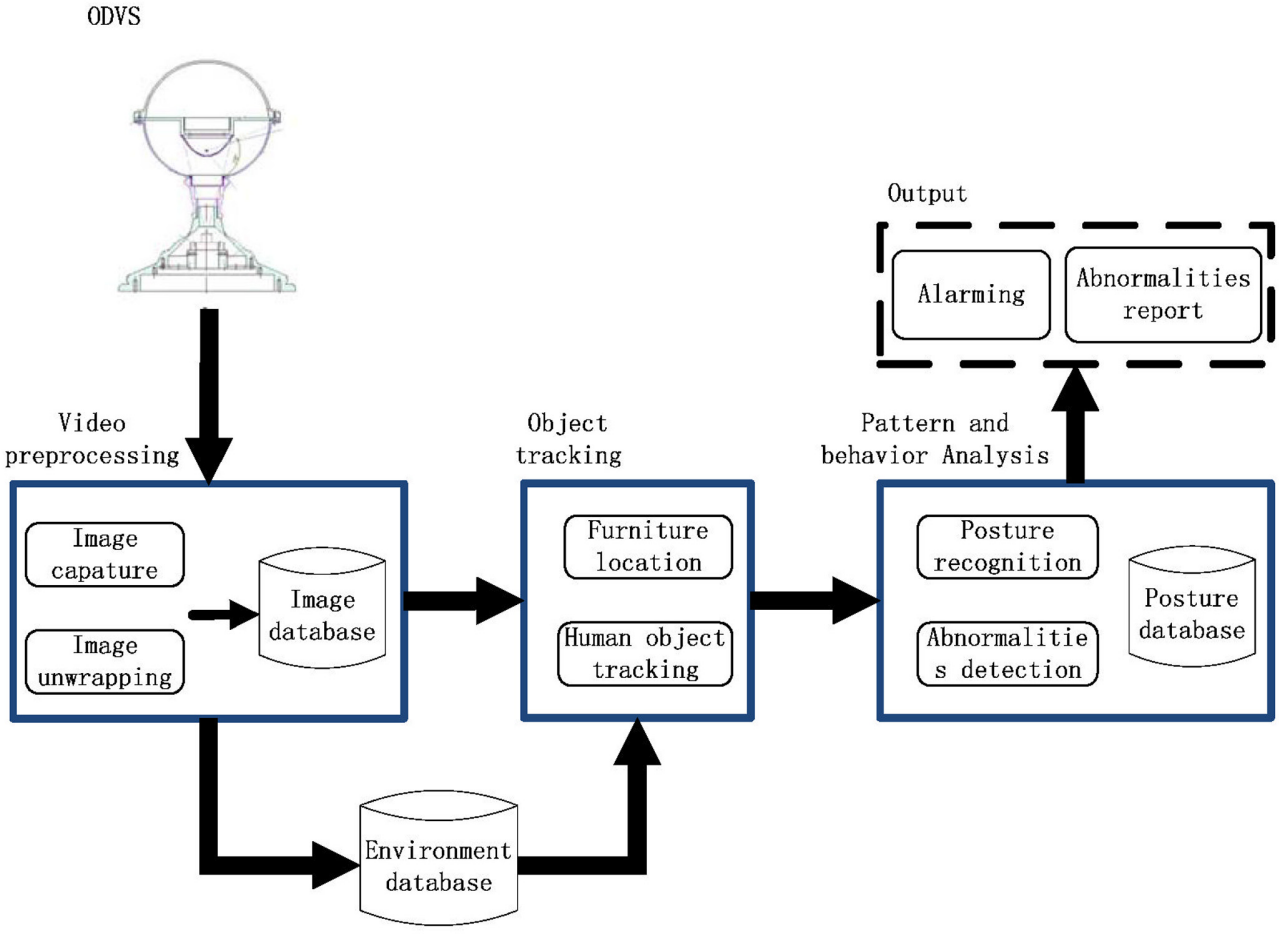
System overview.

Fig 1 describes our system design. The information of elderly persons’ activities can be derived using the panoramic video images via ODVS. After the acquisition of the image sources, they are pre-processed and stored in the database. They are then used to update the background information database, e.g., locations, furniture, and electronic devices etc. The video information is also utilized in the object tracking and pattern recognition algorithms. Combining the posture information and environment information, we can identify the abnormal activities and alarm the appropriate persons when emergency events are detected.

During the development of the system, we are using the existing techniques in many function blocks. The effectiveness and shortcomings of the existing techniques have already been analyzed in the corresponding references. Therefore, we consider it unnecessary and out of the scope of this paper to compare them. Note that we are considering a system with real-world application. Many complicated techniques require extensive training. Thus, their performance is quite dependent on the specific applications. Moreover, sometimes they are unrealistic to be implemented in real-world applications. If for some applications, more complicated and advanced techniques are required, it should be relatively easy to incorporate those techniques into our current system.

### Image Acquisition and Calibration

#### Image Acquisition

**Fig 2:**
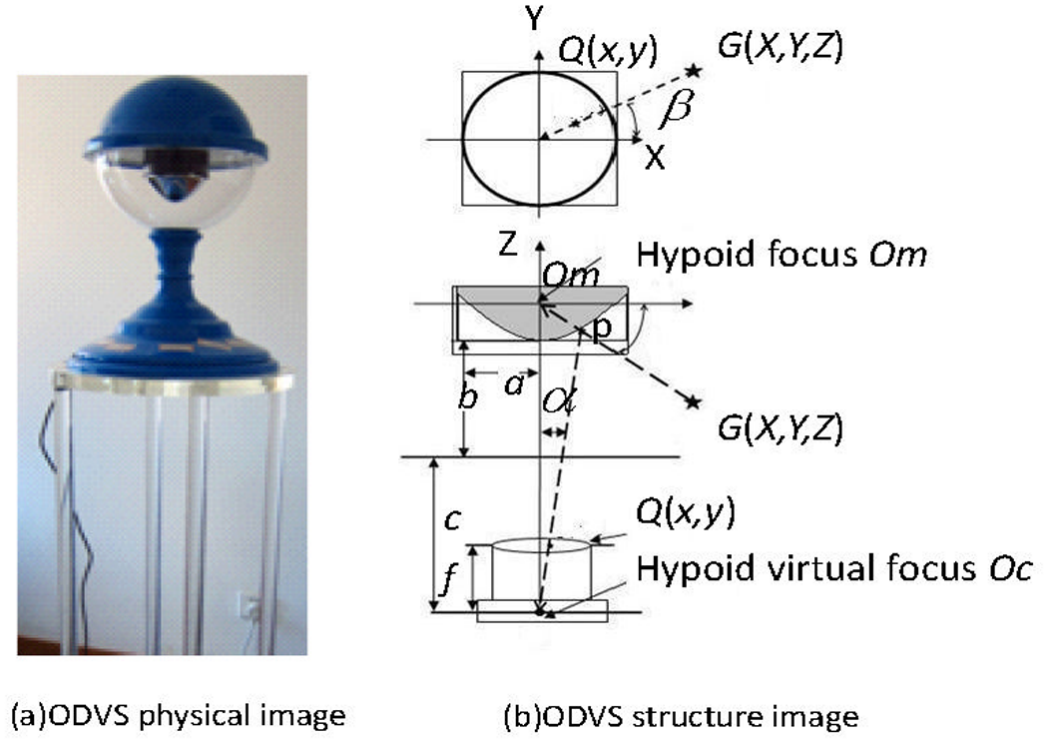
The ODVS device and its structure.

To provide accurate and timely surveillance and care for the elderly persons, monitoring devices should be installed in key locations of human activities. In this work, we assume that elderly persons spend most of their time indoors. The device we used is an ODVS based system. Fig 2 shows the outlook and underlying theory of an ODVS device. A detailed description of the system can be found in Tang et al.’s work [27].

However, one problem for the ODVS system is that the acquired images can not be used for processing directly. The unprocessed image is derived from the mirror plane. We need to convert it to the image plane. Thus, several camera parameters, such as intrinsic geometry, optical property, and 3D coordinate relationship etc., need to be calibrated first [28].

**Fig 3:**
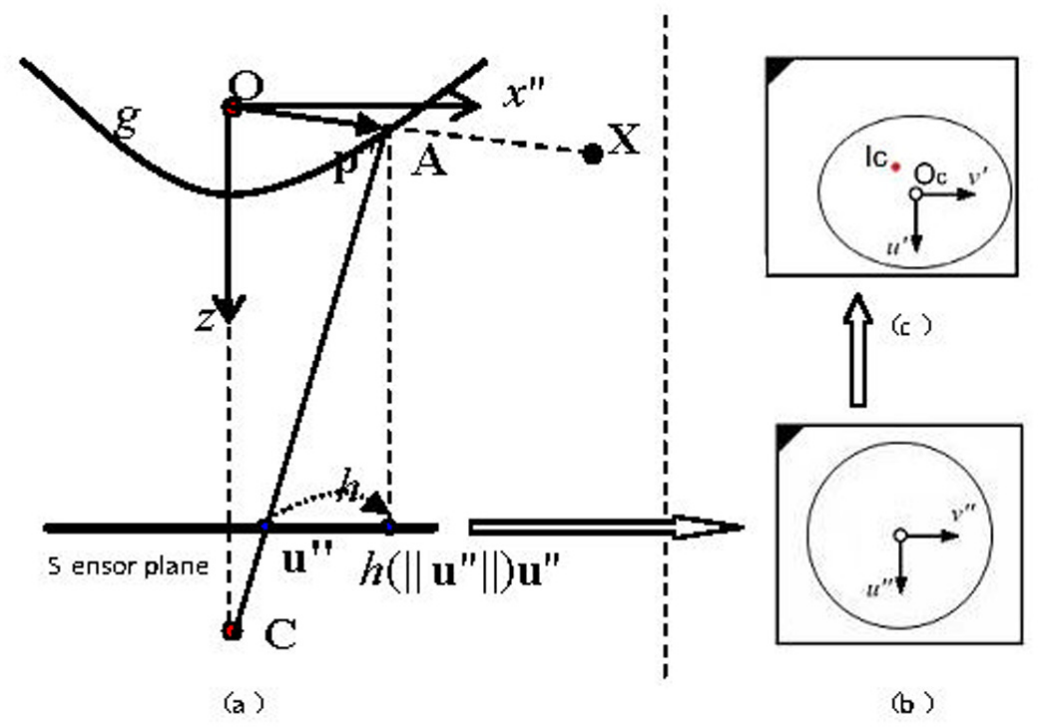
Single view point ODVS model and theory.

Fig 3 shows the theory of our image calibration technique. In the figure, X is a given reference point in the sensor plane and u’ is the corresponding point in the image plane. There is a single view point O. The initial image is acquired in the reflection mirror plane. We need to convert it to the sensor plane first, and then convert it from the sensor plane to the image plane. The image plane is denoted as (u’, v’), the mirror plane is denoted as (u”, v”), and the sensor plane is defined as $\partial r=f(\frac{r^{2}}{dc})\partial \phi$.

In order to compensate the error caused by approximate estimation of the mirror central axis and the camera focus, function *f* is described using Taylor Polynomial as shown in the following equation.

$$\begin{array}{c} f\left(∥u”∥\right)=a_{0}+a_{1}∥u”∥+a_{2}{∥u”∥}^{2}+\cdots +a_{n}{∥u”∥}^{n} \end{array}$$ (1)

By solving the equation, we can calculate the calibrated parameters of the camera. Table 1 shows the calibration results in our experiment.

**Table 1:** ODVS Camera Calibration Results.

Calibration parameters	a_0_	a_2_	a_4_	A	T point	Central accuracy	Calibration
ODVS	-90.37	0.0034	0	-5.22E-05	2.7504	322.75	0.63

#### Image Calibration

It is important to have a precise and detailed description of the locations and environment since the same posture might have completely different interpretations given different circumstances. For example, if an elderly person is lying down, the response should be entirely different depending on he is on the bed or on the floor. Thus, it is important to have an accurate description of the surrounding environment.

**Fig 4:**
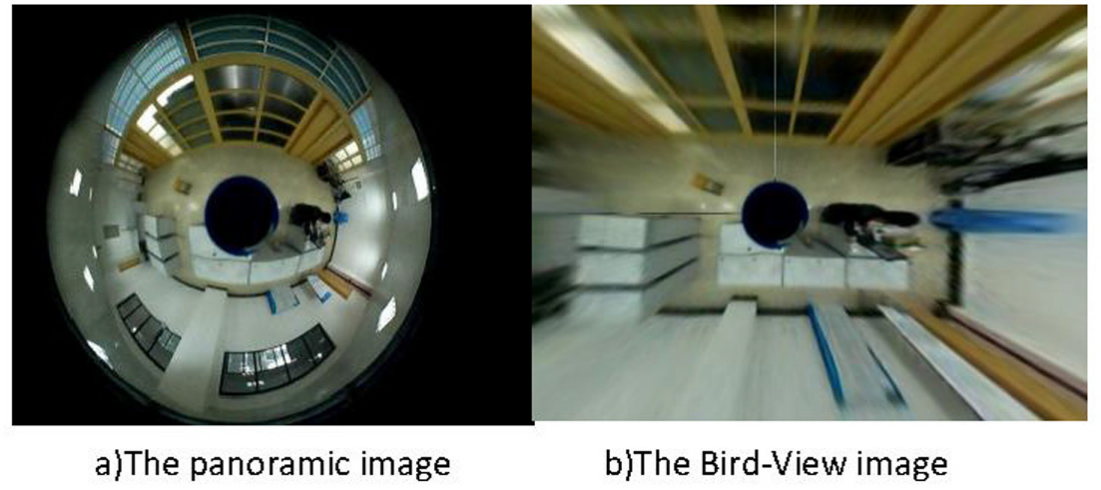
Bird-view transformation of a panoramic image.

However, the problem for the panoramic images is that they typically have significant distortions because of the conversion from the sensor plane to the image plane. Thus, it is hard to generate accurate environment information directly from the panoramic images. To address this problem, we have implemented the bird-view technique, which is a reversal transformation from the image plane to the sensor plane, i.e., physical plane. Fig 4 shows a panoramic image before and after the transformation. It clearly shows that the distortion has been removed after applying the technique.

In our application, most of the indoor objects are still. When the objects are not moving, it becomes extremely difficult to identify and differentiate. Moreover, relying on computer vision to analyze the indoor objects is neither necessary nor feasible. The computation power and prior knowledge required to accurately identify the static environment objects are tremendous. On the other hand, the environment objects information can be easily provided by one’s family members. Thus, we use a pre-calibration based method to locate and identify the indoor objects.

**Fig 5:**
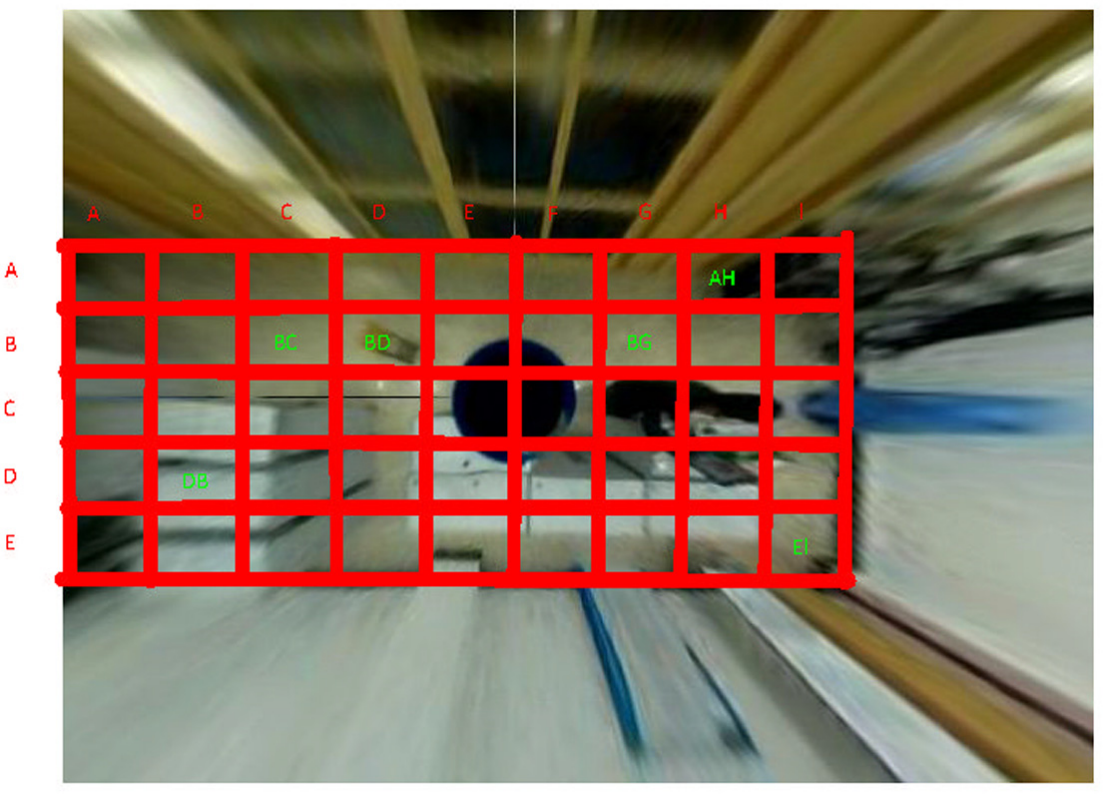
An example of the one-to-one calibration.

Fig 5 shows an example of our pre-calibration method. The physical space of the room is divided into several grids. A one-to-one correlation is established between the foreground objects in the image and the environment elements. The environment elements can then be used to determine the elderly persons’ location and surrounding environment.

In this work, we have implemented the bird-view technique to perform the pre-calibration. Each grid is denoted by its coordinate in the grid. For example, in Fig 5, AH refers to the bathroom entrance; EI refers to the room entrance; BC and BG refer to the ground in the living room; BE refers to the stool in the living room; and DB refers to the desk in the living room. Based on the environment information, we can determine the human behaviors. For example, if the tracking box disappeared after entering grid AG, we can determine that the elderly person has moved into the bathroom.

### Human Object Tracking

#### Tracking Algorithm

To analyze the human behavior automatically, it is important to be able to track the indoor movement of the subjects. There are several existing human subject tacking algorithms, e.g., Meanshift algorithm, Camshift algorithm, feature matching based algorithm, and shape and size based algorithm etc. The common problem for the above algorithms is that they are all very computation-intensive. Thus, they are inadequate to be implemented in real-time monitoring applications. To address this problem, we have developed a motion history or energy images (MHoEI) based technique, which is fast, efficient, and effective

The MHoEI algorithm is developed based on motion history images (MHI) algorithm and motion energy images (MEI) algorithm. For the MHI algorithm, motion history image is acquired using inter-frame difference comparison technique and fixed-interval gray processing technique. The inter-frame difference comparison technique can be quite efficient since typically most of the indoor objects are static. The gray processing technique can create a motion gradient image. It uses the Sobel operator to calculate the orientation and magnitude of the object movement. The MHI updating equation is shown in the following equation.

$$\begin{array}{c} H_{\tau }\left(x,y,t\right)=\{\begin{array}{c} \tau \cdots (D(x,y,t)=1)\\ max(0,H_{\tau }\left(x,y,t-1\right))\cdots \;otherwise, \end{array} \end{array}$$ (2)

where *τ* is the time duration, x and y are different images, D is the motion region of the binary video image sequence, and H is the motion history image. It should be noted that in the MHI algorithm, background modeling is not required, which can significantly reduce the computation cost.

Another motion image acquisition technique is called MEI algorithm, which is the sum of the inter-frame differences. The inter-frame difference is calculated using the binary cumulative motion energy image. The MEI updating equation can be described using the following equation.

$$\begin{array}{c} E_{\tau }\left(x,y,t\right)=⋃D\left(x,y,t-i\right), \end{array}$$ (3)

where is the time duration, D is the motion region of the binary video image sequence, and E is the energy image.

To address the tracking problem of both dynamic and static objects, we propose our MHoEI algorithm. The updating equation of the MHoEI algorithm is defined in the following equation.

$$\begin{array}{c} H_{\tau }\left(x,y,t\right)=\{\begin{array}{c} \tau \;\cdots \;(D(x,y,t)=1)\\ max(0,H_{\tau }\left(x,y,t-1\right))\;\cdots \;\left(S\leq \delta \right)\\ max(0,H_{\tau }\left(x,y,t-1\right)-1)\;\cdots \;otherwise, \end{array} \end{array}$$ (4)

where S is the velocity of the foreground object and *τ* is the duration. The duration can be dynamically adjusted based on the velocity of the foreground object. In general, the time duration is negatively correlated with the velocity, e.g., *τ* decreases when S increases. The gray gradient of foreground object does not change if the velocity is within certain threshold *δ*. Thus, the static foreground objects are remembered.

#### 3D Image Pre-processing

Even after the pre-calibration, it is still inconvenient to process the panoramic images directly using our techniques. As shown in Fig 5, the panoramic image is quite different from the conventional human visions. Thus, we first transform the panoramic images into conventional images using Lin et al.’s unwrapping technique [29].

**Fig 6:**
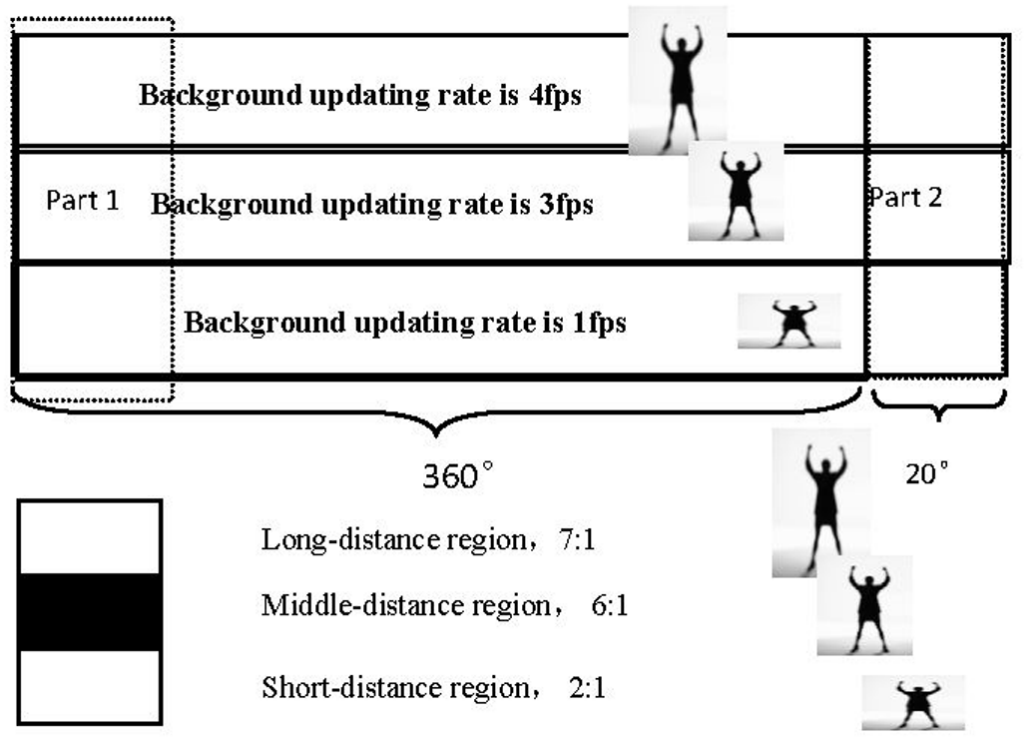
Flow of 380° unwrapping and background updating.

**Fig 7:**
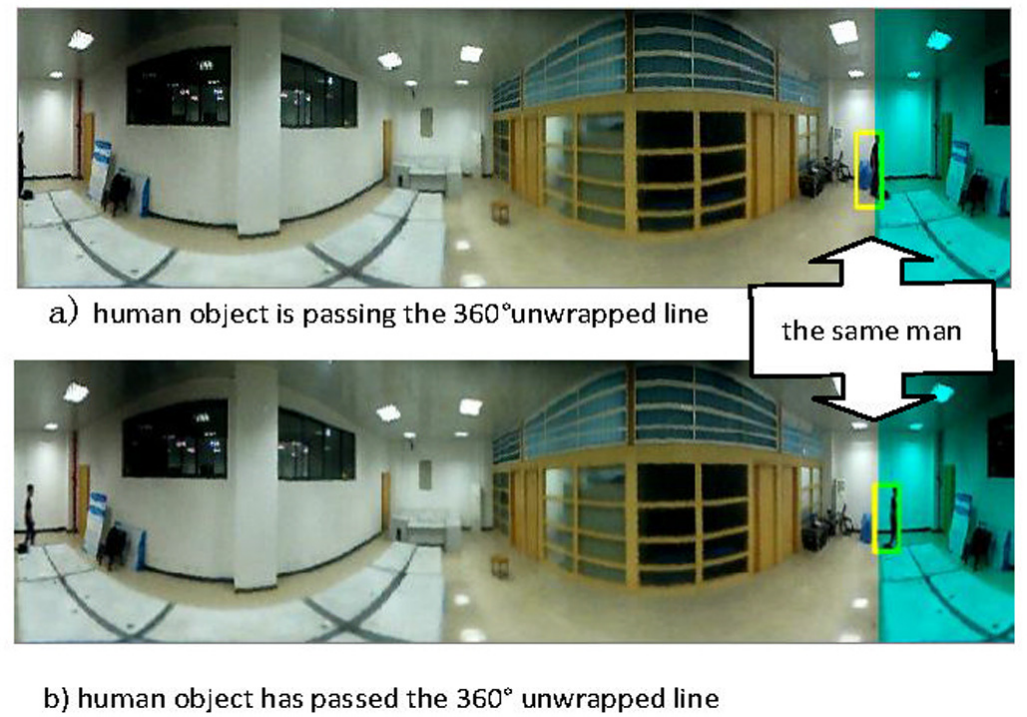
Human object tracking example with 380° unwrapped image.

The MHoEI algorithm is implemented using the 360° unwrapped image. The problem for Lin et al.’s technique is that when the moving target is at the starting edge, i.e., the 0 degree point, it would be recognized as two separate objects. To keep the integrity, accuracy, and continuity of the tracking objects, we have installed a 20 degree overlap region in the unwrapping algorithm. Fig 6 shows the method to perform 380° unwrapping. In the figure, part 1 and part 2 regions contain the same content. Judging by the locations of the object in the image, we can determine whether it is the same object or two separate ones. After the 380 degree unwrapping, the MHoEI algorithm can be implemented on the unwrapped images. Fig 7 shows an example of the object tracking results of the MHoEI algorithm.

The time duration is selected based on the object velocity. However, in the panoramic image, the moving velocity is determined by the distance from the tracking objects to the panoramic image center as well. To accurately estimate the velocity of the moving object, this deviation must be compensated. Moreover, the aspect ratio of the derived human model is also affected by this distance. For example, when the human subject is at the image center, the aspect ratio is unaffected. As one moves further away, the aspect ratio increases accordingly. To address these two problems, we divide the images into different regions based on their distance to the image center, as shown in Fig 6. The velocity and human model aspect ratio are compensated based on the region the human subject is located. For example, the background updating frame number, which is used to determine the velocity, becomes 2, 3, and 4 frames for short, middle, and long range regions, respectively.

### Posture Recognition and Surveillance System

#### Posture Recognition

In this work, we define the human basic posture as standing, sitting, and lying. It should be noted that this definition is just for demonstration and we can easily include new postures in our technique if necessary. Thus, we define P as the set of the three postures as shown in the following equation.

$$\begin{array}{c} P=\{standing,sitting,lying\}. \end{array}$$ (5)

**Fig 8:**
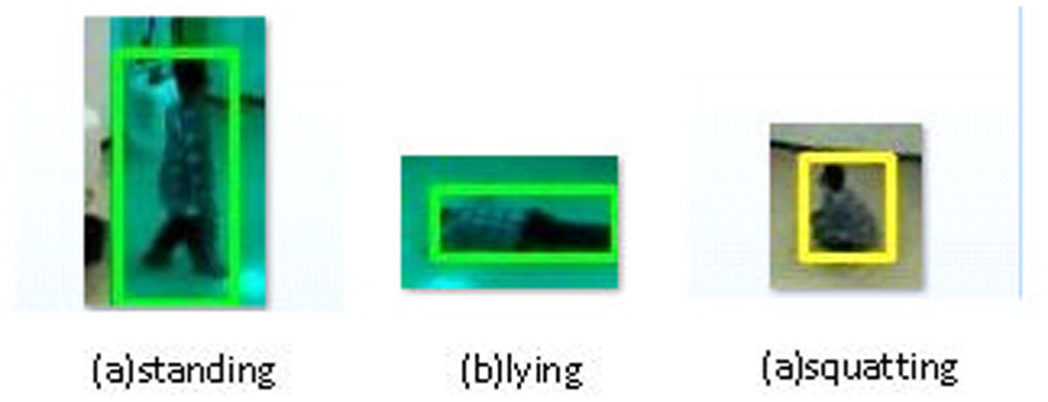
Example of the three human postures.

The image of the human subject is in the 3-D space. To reduce the processing complexity, the original images are transformed into 2-D space first. Then we have implemented the minimum bounding rectangle method to identify the foreground objects. Fig 8 shows an example of human posture recognition. Fig 8 (a), 8 (b) and 8 (c) show the standing, lying, and sitting postures, respectively. The human posture ratio is defined as k = H / W, where H is the height of the bounding rectangle and W is the width. The human posture ratio is then used to analyze the human posture.

The posture is determined by comparing the posture ratio with certain pre-defined threshold. The threshold is derived by statistically analyzing the previous data of the subjectâĂŹs daily life. Table 2 shows the thresholds for different human postures. The thresholds are determined so that the recognition errors are minimized.

**Table 2:** Thresholds of Human Posture Detection.

Posture	Standing	Sitting	Lying
Threshold *k*	*k* ≥ 1.8	1.8 > *k* ≥ 0.7	*k* < 0.7

The problem for the minimum bounding rectangle method is that it has a relatively high recognition error rate when the human actions are not pre-characterized. For example, if the arms of the monitoring subject are expanding, the posture ratio can be changed significantly and thus increase the error rate.

**Fig 9:**
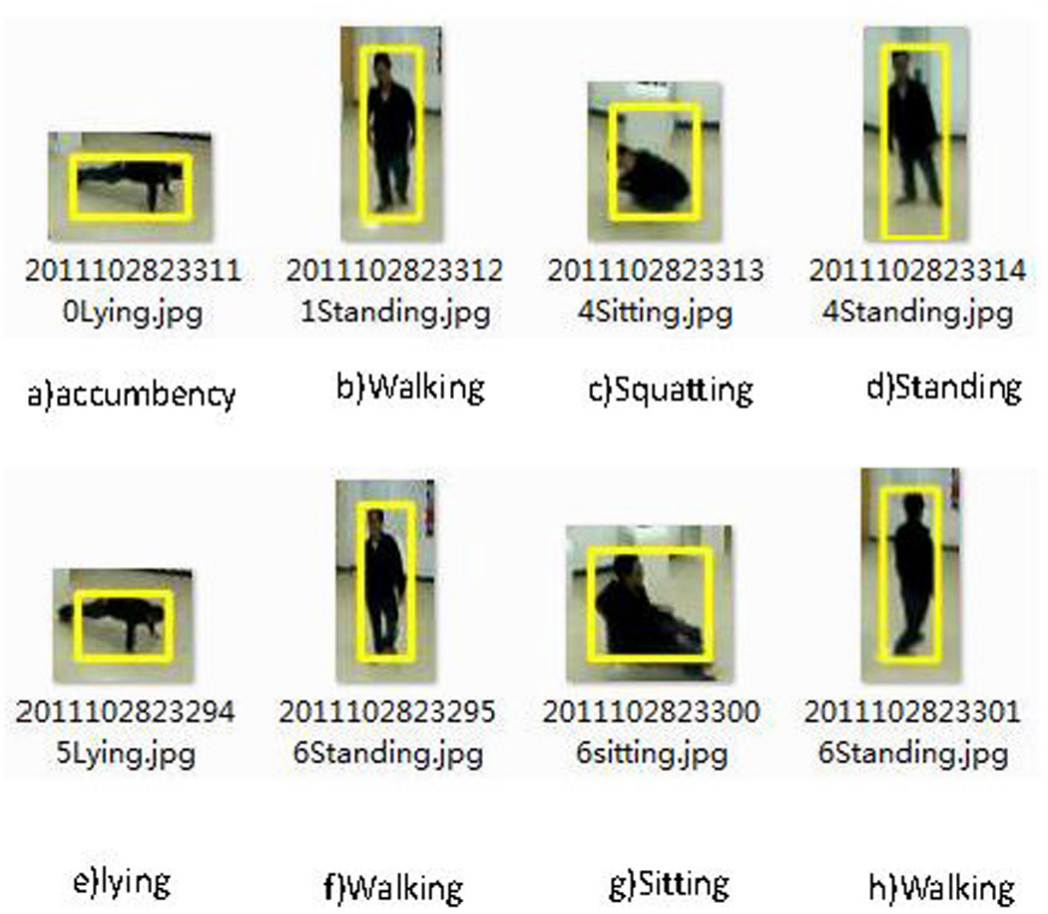
Human posture recognition examples.

To address the human abnormal action problem, we propose to use posture evaluation function. The posture evaluation function is defined using the following equation.

$$\begin{array}{c} S=\frac{\sum F_{g}}{S(T)}, \end{array}$$ (6)

where ∑ *F*_*g*_ = ∑_(*x*,*y*) ∈ *T*_ *I*(*x*, *y*) is the total area of the subject in the image and S(T) is the area of the minimum bounding rectangle. If the subject is in normal state, *F*_*g*_ is close to S(T). In that case, the value of the posture evaluation function is high. On the other hand, if the action of the human subject is unusual, e.g., expanding oneâĂŹs arms, the value of the human posture evaluation function becomes very low. In that way, we can filter out the abnormal actions and increase the posture recognition success rate significantly. Fig 9 shows an example of the human posture recognition results using our technique. It shows that even with complicated actions, our technique can still recognize the human postures accurately.

#### Abnormal Activity Detection

Accurate human posture recognition alone is insufficient for the elderly person monitoring application. The human posture should be further analyzed to detect the abnormal activities. In this work, we combine and analyze different types of data, such as environment, actions, and postures etc., to determine the abnormalities.

Researchers have proposed various techniques to detect the abnormal activities of the elderly persons. For example, the abnormal activities can be detected using the change rate of the human postures [30]. However, in many cases, it is insufficient to use human postures only to detect abnormalities. For different persons, the life styles and habits can differ significantly. Thus, the action rates can also be very different, which make the abnormal detection difficult and inefficient.

To accurately detect the abnormal behavior, we propose to use a modeling and training based technique. The normal behavior pattern is established by training the collected data. By comparing the current human behavior to the trained dataset, the abnormal behavior can be detected if the abnormal degree exceeds certain threshold.

#### Remote Monitoring

Currently, to remotely derive the images of the elderly persons, people use networked camera to acquire and transmit videos. The videos are then manually analyzed by the correspondent persons. There are many problems in this process. For example, the privacy of the individuals can be easily violated, which makes people reluctant to use the system. Moreover, even after compression, transiting the video data back to the server is still slow and expensive. One observation is that most of the daily activities are normal and thus, not interesting to our application. It is much more feasible if we only transmit the scenes with abnormal activities.

To address the problems mentioned above, we employed various techniques. Firstly, instead of recording the individuals directly, we use an agent to replace the elderly person so that the personal characteristics are removed. Secondly, the videos are recorded in the local storage devices and only the generalized behavior codes, such as environment, actions, and behaviors etc., are transmitted through network. Finally, whenever abnormal behaviors are detected locally using our algorithms and technique, the appropriate persons are notified and correspondent videos are transmitted immediately.

To facilitate the remote monitoring application, a website is designed specifically. Family members and caring persons can access the processed videos easily online. The website is embedded with an encrypted database which stores the history monitoring information for each individual. The history information can be used to analyze the behavior and activity patterns later.

## Results

### Experiment Setup

The safety monitoring system is implemented using Java and C++. The system platform is run on a computer with Windows XP, 3G memory and 2.7G Pentium 4 CPU. Self-developed ODVS devices are deployed. The resolution of the panoramic video images is set at 640 × 480 dpi. The resolution of unwrapped image is 740 × 180 dpi.

The experiment is conducted in an 80m^2^ conference room. The ODVS device is located in the center of the room and its height is about 1.8 m. One of the authors is moving and performing various actions in the room, in simulation of an elderly person. During the experiment, we have recorded 4 sets of videos. In the first video set, a person moves around the ODVS in circle. Thus, we can test the performance of the system while the object moving across the unwrapping line. In the second video set, the human object is performing three different posture, which are standing, sitting, and lying. In the third video set, there are many colorful objects in the scene. Therefore, we can test the algorithms in the situation of blocking, shading, and similar background color etc. In the last video set, the human object performs various motion pattern, e.g., sudden still from motion etc.

Besides the local hardware infrastructure, the safety monitoring network is also developed. The local information can be transferred back to the central server through internet.

### Human Object Tracking

**Fig 10:**
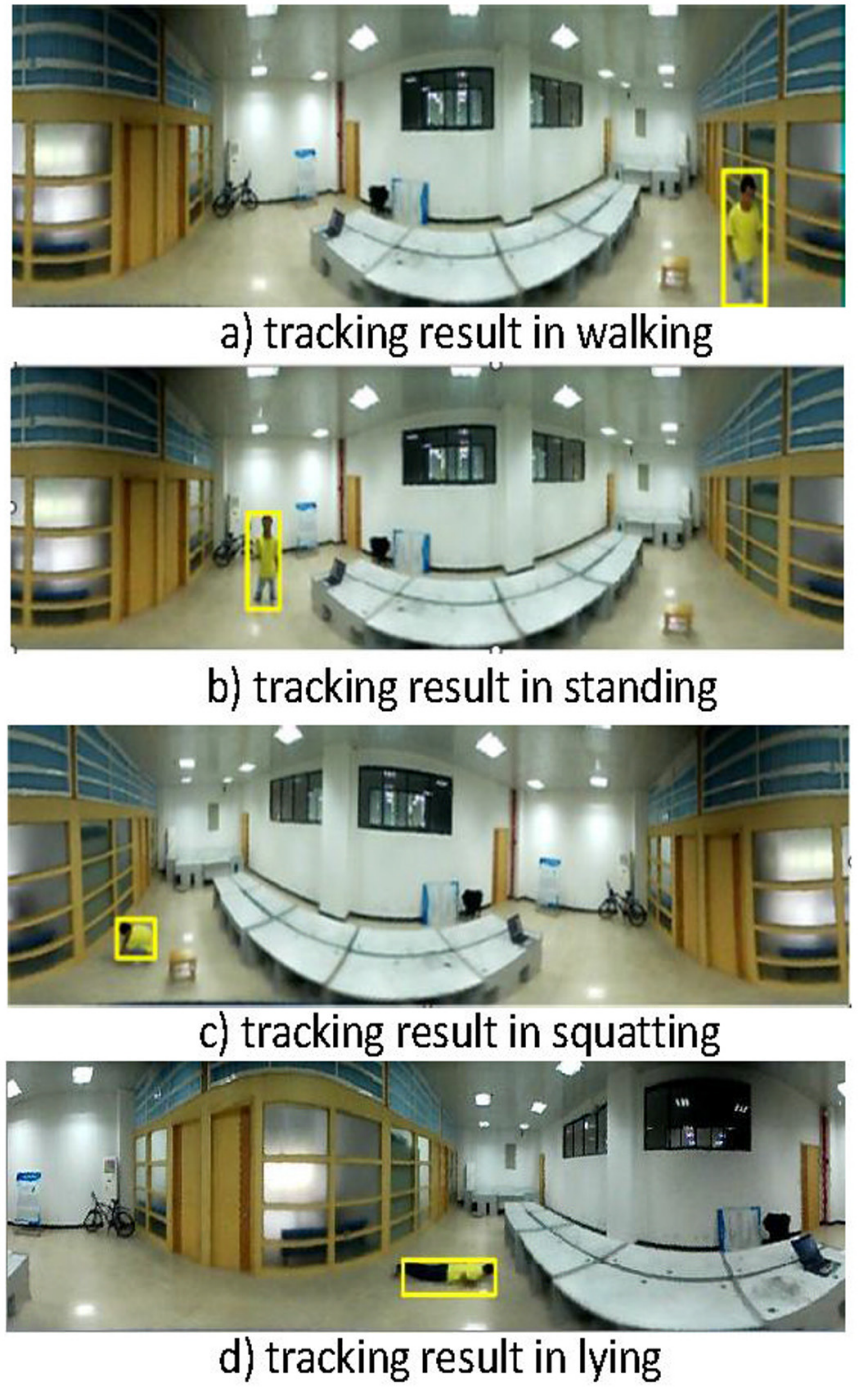
Comparison example of the human tracking algorithms.

Human object tracking is critical to automatically identify the human behaviors. To demonstrate the effectiveness and accuracy of our MHoEI algorithm, we have performed a comparing experiment. In this work, we compare our technique with the Cam-shift algorithm. The result is shown in Fig 10.

The experimental results show that the motion target can be tracked steadily using the MHoEI algorithm. Moreover, because of the effect of the shadows, our technique can have a much higher identification quality than the Cam-shift algorithm. The average tracking error of our technique is less than 7 pixels.

Because of the tracking algorithm is performed in real-time, computation requirement is very important. Thus, we have compared the running time of our algorithm and the Cam-shift algorithm. The video is streamed at 10 frame per second and the results are shown in Tables 3 and 4.

**Table 3:** Human Object Extraction Algorithm Efficiency Comparison.

Algorithms	Time (s)	Frame	Frame size (dpi)	Process time (per frame)
Our algorithm	2.65	123	740×180	0.0216
Camshift	3.43	100	740×180	0.0343

**Table 4:** Human Object Tracking Algorithm Efficiency Comparison.

Algorithms	Time (s)	Frame	Frame size (dpi)	Process time (per frame)
Our algorithm	3.74	111	740×180	0.034
Camshift	9.22	190	740×180	0.049

In this work, we are considering processing time (for embedded applications), performance, and system cost simultaneously. To allow the system running on the low-cost embedded platforms, such as a TI DSP core, it is required that the tracking algorithms to be simple and effective. Since the recognition performance is already acceptable (98.6% identification rate), we are more focused on the running time of the algorithms. We have compared our algorithm with a most commonly used algorithm called Camshift, and the result is shown

It should be noted that for the existing light weight object tracking algorithms, including Camshift, there is a problem that they can not track the static objects. Our MHoEI based algorithm, which is a hybrid algorithm of MH and EI, can track static objects, and have improved efficiency as well.

Experimental results show that our algorithm can reduce the computational cost significantly. The running time per frame is improved by 58.5%, which can greatly reduce the workload and processing time.

### Posture Recognition Evaluation

**Fig 11:**
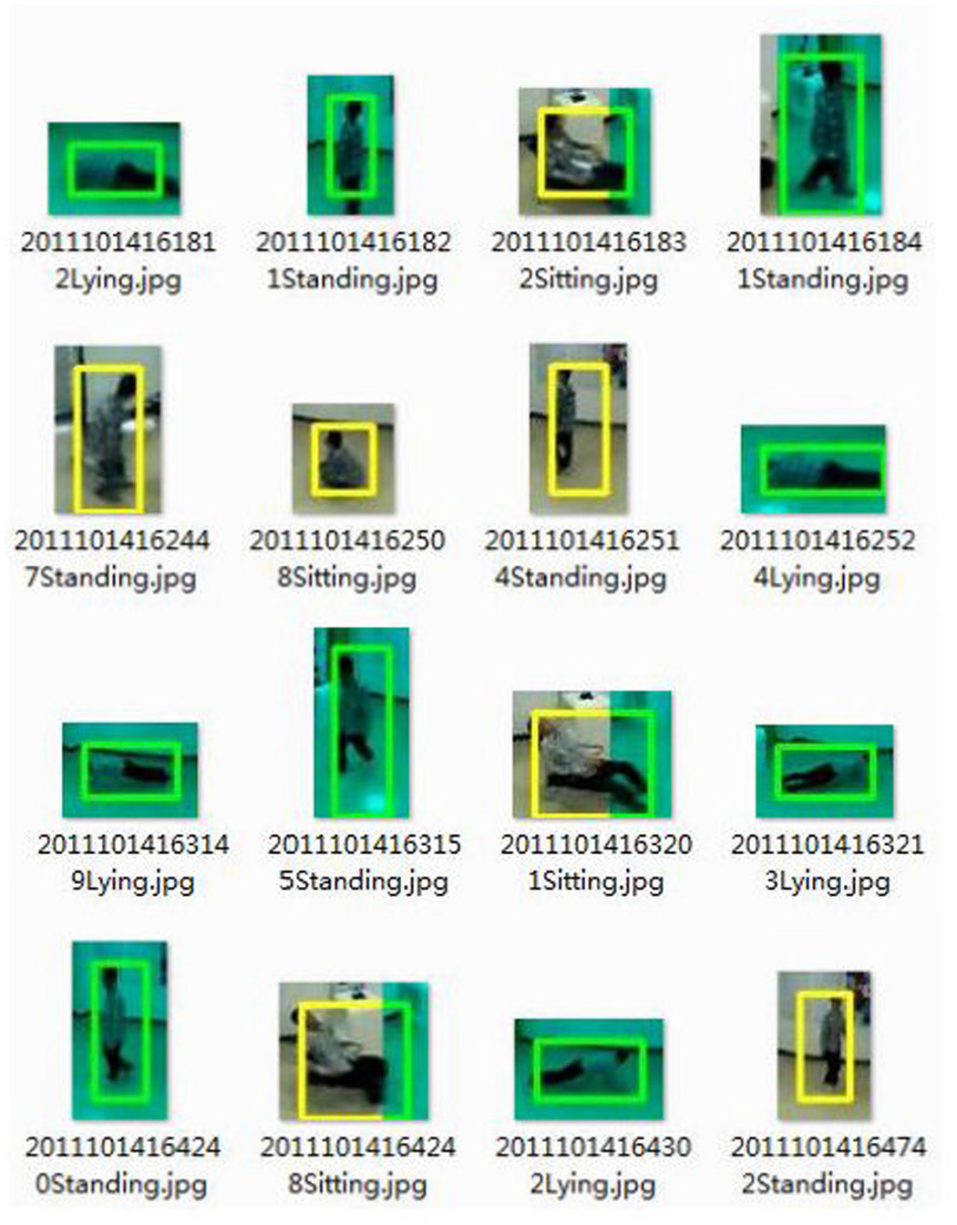
Experiment results of our posture recognition technique.

To evaluate our posture recognition technique, we have conducted an experiment in the information center of the university. The experiment is performed by co-authors of the paper. They are required to perform various actions and their postures are analyzed in real-time. Fig 11 shows some example results. It demonstrates that our technique can differentiate the postures effectively.

Table 5 shows the identification results of our posture recognition technique. During the experiment, the system is required to differentiate 4,000 postures. There are only 55 misjudgments for the lying posture and the average success rate reaches 98.6%. The reason for the errors is that when people are lying towards the ODVS directly, the minimum bounding rectangle becomes similar to the sitting posture. In that case, lying posture can be recognized as sitting mistakenly. However, this scenario is quite rare and can be addressed by comparing the current frame with the history.

**Table 5:** Posture Recognition Experiment Results.

Postures	Environment	Total	True	False	Accuracy
Standing	Indoor	1450	1450	0	100%
Standing	Indoor	1320	1320	0	100%
Standing	Indoor	1230	1175	55	95.52%

## Discussion

Intelligent surveillance is an important research topic in the nursing and home-care robotic applications. In this work, we have proposed a safety monitoring system for elderly persons using ODVS, which can provide 360° panoramic image. In this work, we have developed calibration algorithm to remove the panoramic image distortions and map the image pixels to the physical location. To track the locations of the individuals, we have proposed a MHoEI based algorithm to indentify both the moving and still objects. A human aspect ration based posture recognition technique is also developed. Besides the local surveillance hardware and system, we have also designed the network to transmit the collected data.

## References

[1] Tang YP, Wang W, Fu YZ. Elder Health Status Monitoring through Analysis of Activity. Chinese Journal of Computer Engineering and Applications. 2006;43(3):211–213.

[2] Zhou Z, Dai W, Eggert J, Giger J T, Keller J, Rantz M, et al A real-time system for in-home activity monitoring of elders. Proceedings of International Conference of the IEEE Engineering in Medicine and Biology Society. 2009;6115–6118. 10.1109/IEMBS.2009.5334915 19965263

[3] Paul C, Meena G, Catherine G, Jenny W. Remote Monitoring and Adaptive Models for Caregiver Peace of Mind. Proceedings of 2003 International Conference on Aging, Disability and Independence. 2003;183–184.

[4] Auvinet E, Multon F, Saint-Arnaud A, Rousseau J, Meunier J. Fall detection with multiple cameras: an occlusion-resistant method based on 3-d silhouette vertical distribution. IEEE Trans Inf Technol Biomed. 2011;15(2):290–300. 10.1109/TITB.2010.2087385 20952341

[5] Andrew S, Neil J. Smart Sensor to Detect the Falls of the Elderly. IEEE Pervasive Comput. 2004;3(2):42–47. 10.1109/MPRV.2004.1316817

[6] Yilmaz A, Javed O, Shah M. Object tracking a survey. ACM J Comput Surv. 2006;38(4):1–46.

[7] Kass M, Witkin A, Terzopoulos D. Snakes active contour models. Int J Comput Vis. 1988;1(4):321–331. 10.1007/BF00133570

[8] Xu C, Yezzi A, Prince JL. On the relationship between parametric and geometric active contours. Asilomar Conf Signals Syst Comput. 2015;483–489.

[9] Jang A, Choi K, Toh K, Teoh A, Kim J. Object tracking based on an online learning network with total error rate minimization. Pattern Recognition. 2015;48(1):126–139. 10.1016/j.patcog.2014.07.020

[10] Li WY, Wang P, Jiang R, Qiao H. Robust object tracking guided by top-down spectral analysis visual attention. Neurocomputing. 2014.

[11] Ji R, Duan LY, Chen J, Yao H, Yuan J, Rui Y, et al Location discriminative vocabulary coding for mobile landmark search. Int J Comput Vis. 2012;96(3):290–314. 10.1007/s11263-011-0472-9

[12] Ji R, Yao H, Liu W, Sun X, Tian Q. Task-dependent visual-codebook compression. IEEE Trans Image Process. 2012;21(4):2282–2293. 10.1109/TIP.2011.2176950 22128004

[13] Ji R, Duan LY, Yao H, Xie LX. Learning to Distribute Vocabulary Indexing for Scalable Visual Search. IEEE Transactions on Multimedia. 2012.

[14] Guan T, He Y, Gao J, Yang J, Yu J. On-Device Mobile Visual Location Recognition by Integrating Vision and Inertial Sensors. IEEE Transactions on Multimedia. 2013;15(7):1688–1699. 10.1109/TMM.2013.2265674

[15] Guan T, He Y, Duan LY, Yu J. Efficient BOF Generation and Compression for On-Device Mobile Visual Location Recognition. IEEE Transactions on Multimedia. 2014;21(2):32–41. 10.1109/MMUL.2013.31

[16] Guan T, Duan LY, Yu JQ, Chen YJ, Zhang X. Real Time Camera Pose Estimation for Wide Area Augmented Reality Applications. IEEE Computer Graphics and Application. 2011;31(3):56–68. 10.1109/MCG.2010.23 24808092

[17] Taylor ME, Ketels MM, Delbaere K, Lord SR, Mikolaizak AS, Close JCT. Ait Impairment and Falls in Cognitively Impaired Older Adults: An Explanatory Model of Sensorimotor and Neuropsychological Mediators. Age and Ageing. 2012;41(5):665–669. 10.1093/ageing/afs057 22572239

[18] Wang ML, Huang CC, Lin HY. An intelligent surveillance system based on an omnidirectional vision sensor. Proceedings of the IEEE Conference on Cybernetics and Intelligent Systems. 2006;1–6.

[19] Han JH, Jeon HS, Park KS. Gait detection from three dimensional acceleration signals of ankles for the patients with Parkinson’s Disease. ITAB. 2008.

[20] Yoshida T, Mizuno F, Hayasaka T, Tsubota K, Wada S, Yamaguch T. Gait analysis for detecting a leg accident with an accelerometer. Proceedings of the Transdisciplinary Conference on Distributed Diagnosis and Home Healthcare. 2006 10.1109/DDHH.2006.1624793

[21] Kim SH, Kim DW. A Study on Real-Time Fall Detection Systems Using Acceleration Sensor and Tilt Sensor. Sens Lett. 2012;10:5–6.

[22] Yoo J, Yan L, Lee S, Kim H, Yoo H. A wearable ECG acquisition system with compact planar-fashionable circuit board-based shirt. IEEE Trans Inf Technol Biomed. 2009;13(6):897–902. 10.1109/TITB.2009.2033053 19789119

[23] Hynes M, Wang H, Kilmartin L, McCarrick E. Intelligent video surveillance for monitoring elderly in home environments. International Conference on Consumer Electronics. 2010.

[24] Nasution A, Emmanuel S. Gait detection from three dimensional acceleration signals of ankles for the patients with Parkinson’s Disease. IEEE Workshop Multimed Signal Proc. 2007.

[25] Foroughi H, Aski BS, Pourreza H. Intelligent video surveillance for monitoring fall detection of elderly in home environments. Int Conf Computer and Information Technology. 2008;219–224.

[26] Lin HY, Wang ML, Huang CC, Tsai BW. Intelligent surveillance using an omnidirectional CCD camera. Automatic Control Conference. 2005.

[27] Tang YP, Ye YJ, Zhu YH, Gu XK. The application research of intelligent Omni-directional Vision Sensor. Chinese Journal of Sensors and Actuators. 2007;20(6):1316–1320.

[28] MicksiK B. Two-View Geometry of Omnidirectional Camera. Ph.D. Thesis Czech Technical University in Prague. 2004.

[29] Scaramuzza D, Siegwart R. A Practical Toolbox for Calibrating Omnidirectional Cameras. Vision Systems: Applications, 2007;297–310.

[30] Tang YP, Jin SJ, Yang ZY, You SS. Detection Elder Abnormal Activities by using Omni-directional Vision Sensor: Activity Data Collection and Modeling. International Joint Conference of SICE-ICASE. 2006;3850–3853.

